# Algorithm based smartphone apps to assess risk of skin cancer in adults: systematic review of diagnostic accuracy studies

**DOI:** 10.1136/bmj.m645

**Published:** 2020-02-25

In this paper by Freeman and colleagues (*BMJ* 2020;368:m127, doi:10.1136/bmj.m127, published 10 February 2020), the app named “SkinScan” in the 2014 Chadwick et al study is a predecessor of the SkinVision app and not related to the currently available CE marked TeleSkin “skinScan” app as is implied. Thus the abstract should state: “No published peer reviewed study was found evaluating the TeleSkin skinScan app. SkinVision was evaluated in three studies (n=267, 66 malignant or premalignant lesions)”. The results should state: “No published peer reviewed study was found evaluating the TeleSkin skinScan app. The SkinVision predecessor app SkinScan was evaluated in a single study of only 15 lesions (five melanomas).^23^” The skinScan row in table 1 should report high risk as “Atypical” and the first footnote in table 1 should state: “*This is the TeleSkin skinScan app and not the SkinScan app evaluated by Chadwick and colleagues.^23^ No published peer reviewed study was found evaluating the TeleSkin skinScan app.” The text describing table 1 should state that two apps, skinScan and SpotMole, do not provide a moderate risk result, that MelApp does not recommend an action following a high risk result, and that only Mole Detective recommends monitoring after a moderate risk result. Table 2 and figure 2 should state: “The Chadwick 2014 SkinScan app is a predecessor of SkinVision and not related to the CE marked TeleSkin skinScan app.” 

Under the heading “Misleading regulation” in the linked editorial by Morley and colleagues (*BMJ* 2020;368:m428, doi:10.1136/bmj.m428, published 10 February 2020), the second sentence should state: “Freeman and colleagues found no peer reviewed, published studies evaluating the Teleskin skinScan app.”

The research paper and linked editorial will be updated in due course. 

